# Efficacy and safety of mesenchymal stem cell therapy for ovarian ageing in a mouse model

**DOI:** 10.1186/s13287-024-03698-0

**Published:** 2024-04-03

**Authors:** Wendi Pei, Lin Fu, Wenhuan Guo, Yibo Wang, Yong Fan, Rui Yang, Rong Li, Jie Qiao, Yang Yu

**Affiliations:** 1 Center for Reproductive Medicine, Department of Obstetrics and Gynecology, Beijing Key Laboratory of Reproductive Endocrinology and Assisted Reproductive Technology and Key Laboratory of Assisted Reproduction, Ministry of Education, Peking University Third Hospital, Beijing, 100191 China; 2 Clinical Stem Cell Research Center, Peking University Third Hospital, Beijing, 100191 China; 3 State Key Laboratory of Female Fertility Promotion, Peking University Third Hospital, Beijing, 100191 China; 4 Key Laboratory for Major Obstetric Diseases of Guangdong Province, The Third Affiliated Hospital of Guangzhou Medical University, Guangzhou, 510150 China

**Keywords:** Mesenchymal stem cell, Ovary, Ageing, Mouse model, Safety

## Abstract

**Background:**

Ovarian ageing is one of the major issues that impacts female fertility. Mesenchymal stem cell (MSC)-based therapy has made impressive progress in recent years. However, the efficacy and safety of MSCs, as nonautologous components, remain to be further verified.

**Methods:**

Two common sources of MSCs, umbilical cord-derived MSCs (UC-MSCs) and adipose tissue-derived MSCs (AD-MSCs), were orthotopically transplanted into a mouse model of ovarian ageing to evaluate their therapeutic effects. The safety of the treatment was further evaluated, and RNA sequencing was performed to explore the underlying mechanisms involved.

**Results:**

After orthotopic transplantation of MSCs into the ovary, the oestrous cycle, ovarian weight, number and proportion of primary follicles, granulosa cell proliferation, and angiogenesis were improved. The effects of AD-MSCs were superior to those of UC-MSCs in several indices, such as post-transplant granulosa cell proliferation, ovarian weight and angiogenesis. Moreover, the tumorigenesis, acute toxicity, immunogenicity and biodistribution of MSCs were evaluated, and both AD-MSCs and UC-MSCs were found to possess high safety profiles. Through RNA sequencing analysis, enhancement of the MAPK cascade was observed, and long-term effects were mainly linked to the activation of immune function.

**Conclusions:**

Orthotopic transplantation of MSCs displays significant efficacy and high safety for the treatment of ovarian ageing in mice.

**Supplementary Information:**

The online version contains supplementary material available at 10.1186/s13287-024-03698-0.

## Background

Mesenchymal stem cells (MSCs) represent a type of adult stem cell that originates from the mesoderm, with strong self-renewal capacity as well as multidirectional differentiation potential [1]. MSCs can be derived from various tissues, including the umbilical cord, umbilical cord blood, placenta, periodontal ligament, fat tissue and bone marrow [2]. Since it is relatively easy to obtain and expand MSCs, the multiple functions of MSCs, such as nutrient provision, homing/migration regulatory function, and immune regulatory competence, are gradually being recognized and summarized [3]. Currently, MSCs have emerged as a promising new and efficient therapeutic option for various diseases, especially those considered refractory to traditional methods [4].

Physiological reproductive ageing is attributed to a decrease in both the quantity and quality of oocytes within the follicles of the ovarian cortex [5]. Endocrine, paracrine, genetic, and metabolic factors are believed to be influential, although the causal mechanisms behind the decline in oocyte pool and follicle quality remain under investigation [6, 7]. As women's education, social participation, and the availability of contraceptive methods increase, the need to have children is being postponed to an older age by an increasing number of women [8]. However, women's fertility declines with age, and the delayed age of motherhood has led to an increase in the number of patients diagnosed with female infertility and utilizing assisted reproductive technologies [9]. The process of female reproductive ageing is predominantly attributed to changes in ovarian function [10]. Ovarian ageing, which leads to ovarian failure and menopause, is a continuous process. The ovaries are more susceptible to the effects of natural ageing than other organs and tissues, making ovarian ageing an urgent clinical issue to be addressed. Treatments for ovarian ageing include growth hormone therapy, antioxidant supplements such as melatonin and vitamin C, mitochondrial therapy, and personalized treatments tailored to each patient with their special fertility problems [11–13]. MSC therapy, as a new antiageing therapy, is currently in the preliminary stage of clinical application in the human ovary [14, 15]. Previous studies have mainly used autologous-derived MSCs and focused on women with premature ovarian failure and ovarian hypo-responsiveness. As a cell product that can be produced industrially in large quantities, if the efficacy and safety of allogeneic sources can be fully established, MSCs have the potential to greatly improve the feasibility of clinical applications, resulting in major benefits to a greater number of women with ovarian ageing.

Before MSCs can be used as a routinely available clinical treatment, stringent medicinal level tests and identifications are needed for their application safety. However, previous studies were mainly based on single-source-derived MSCs in rat or mouse models, and only a few indicators were tested. Therefore, the efficacy and safety of MSC ovarian therapy have not been adequately and systematically evaluated.

**Fig. 1:**
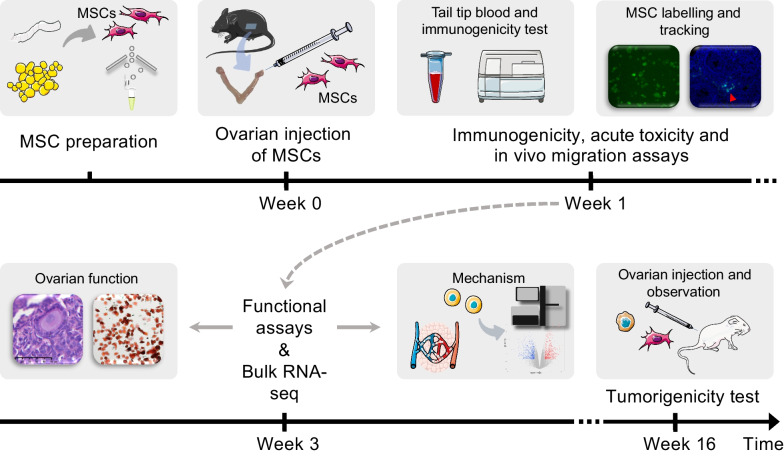
Flowchart overview of the evaluation of the efficacy and safety of MSC therapy

In this study, MSCs were extracted from umbilical cords and adipose tissue, which are commonly considered “waste” tissues in clinical settings, and were cultivated under GMP-grade conditions in compliance with production and safety regulations [16, 17]. The cells were then injected into different mouse models for efficacy and safety verification. For validity verification, estrusthe oestrus cycle, ovarian weight, total/split follicle number and ratio, proliferative status and neovascularization were monitored. For safety, tumorigenicity, acute toxicity (body weight, organ pathology, etc.), immunogenicity (cellular and molecular), and in vivo migration assays were performed. Furthermore, RNA sequencing of mouse ovaries after MSC transplantation was performed. The flowchart overview of this study is shown in Fig. 1.

## Materials and methods

### Animals

Female C57BL/6 mice of different ages (4 weeks old for the test of acute toxicity, 8 weeks old for the observation of in vivo migration, 9 months old for immunogenicity tests, 4–5 months old and 10–12 months old were set as the young group and the naturally ageing group, respectively, for the test of efficacy after MSC treatment) and female BALB/c nude mice (4–6 weeks old for the tumorigenicity test) with reliable health and immune records were purchased from Beijing Vital River Laboratory Animal Technology Co., Ltd., and housed under SPF conditions with temperature control (22 ± 1 °C), humidity control (60 ± 10%), a 12 h light/12 h dark cycle, ad libitum water and conventional mouse chow. A total of 119 C57BL/6 mice and 48 BALB/c nude mice were used. Mice with no apparent abnormalities in appearance or behaviour were selected and allocated to groups randomly using a random number generator. The order of treatments and measurements for each mouse was randomly assigned. During the outcome evaluation and data analysis stages, the group allocation information was made known. Throughout the entire experimental period, the body weight, appearance and behaviour of mice in each group were monitored weekly. Our reporting of animal experiments adheres to the ARRIVE guidelines.

### MSC preparation and characterization

Clinical-grade AD-MSCs and UC-MSCs were isolated from the fat tissue of healthy donors during liposuction surgery and full-term umbilical cord tissue that was free of any clinical disorders after neonatal delivery, respectively. The tissues were collected, stored aseptically on ice and processed by the isolation method under the GMP standard within 4 h of delivery.

Briefly, the adipose tissue samples were washed in PBS, minced with a sterile scalpel, and plated in polypropylene culture flasks. After adherence, serum-free medium (NC0103, NC0103.S, Yocon) for MSCs was added. When MSCs sprouted from the tissue fragments, the adipose tissue was removed, and the medium was replaced. For umbilical cord tissue processing, the tissue was sterilized in 75% ethanol for 10 s and rinsed with 0.9% sodium chloride to remove surface bloodstains and drain blood from the vessels. The tissue was then cut open along the extension axis, and the two arteries and one vein inside it were separated and discarded. After three washes with 0.9% sodium chloride, the tissue was cut into small fragments of approximately 1–2 mm^3^. Next, the fragments were evenly inoculated into 75 T cell culture flasks, with approximately 100–120 pieces per flask. Complete culture medium was then added to the flask, and it was inverted and incubated under 5% CO_2_ at 37 °C and turned over after 4 h. Finally, the cells were observed daily under an inverted microscope, and P0–P1 passaging was performed when the cell confluence reached 40–60% on Days 9–13.

After reaching approximately 80% confluence, the MSCs were harvested using MSC-specific digestive enzymes (NC1004.1, Yocon), passaged and seeded at a density of 15,000 cells per cm^2^ or cryopreserved with serum-free cryopreservation medium. For surface labelling analysis, no less than 2*10^6^ cells were used, and FITC or phaeohemoglobin binding anti-CD11b, anti-CD19, anti-CD34, anti-CD44, anti-CD45, anti-CD73, anti-CD90 and anti-CD105 was performed via flow cytometry (BD FACSAria III, USA) (Additional file 1: Figure S1). For the tumorigenicity test, MSCs from P10 were used, and for the other experiments, MSCs from P3–5 were used.

### Tumour cell culture

The ovarian cancer cell line ES-2 (iCell-h060, iCell) was used as a positive control for tumorigenicity detection. The cells were cultured with McCoy’s 5A full medium (16600082, Gibco) containing 10% foetal bovine serum (10270106, Gibco) and 1% penicillin‒streptomycin solution (15140122, Gibco). After reaching approximately 80% confluence, the cells were digested with 0.25% trypsin and seeded at a density of 15,000 cells per cm^2^. All culture flasks were maintained in incubators at 37 °C and a humidified atmosphere of 5% CO_2_.

### MSC transplantation

For the therapeutic effects study, acute toxicity test, immunogenicity study and biodistribution study, a single-dose bilateral ovarian injection with 3.5*10^5^ MSCs per ovary in 10 µl was performed. For the tumorigenicity test, all groups received a single-dose bilateral ovarian injection with 1*10^6^ cells per ovary in 10 µl. Briefly, the mice were anaesthetized using a ventilator (R500IE, RWD), with 2.5% isoflurane for pre-anaesthesia and 1.5–2% isoflurane for maintaining anaesthesia, and their skin was prepared under anaesthesia. Subsequently, an incision of approximately 0.5 cm was made 1 cm away from the spot 1 cm below the highest point of spinal curvature on the left and right sides using ophthalmic scissors. The subcutaneous tissue was bluntly separated using scissors. Once the muscle layer was fully exposed, an incision of approximately 0.5 cm was made, and the ovaries were gently pulled out using ophthalmic forceps. Ten microlitres of fluid was aspirated with a microsyringe and injected into the ovary, and the syringe was gently pushed to complete the injection. Afterwards, the ovaries were carefully placed back with forceps, and sutures were made layer by layer. Finally, the mice were sterilized, removed from the respiratory anaesthesia machine and placed on a heating pad until they awoke.

### Shorr staining

Vaginal smears were obtained from mice daily at 9:00 am for eight days and stained with Shorr staining methods to observe the oestrous cycle in each group. The oestrous cycle of mice consists of three sequential stages as follows: proestrus, estrus, and metestrus/diestrus, which were identified based on the presence or absence of cornified epithelium, nucleated epithelial cells, and leukocytes [18].

### Sample collection

Before euthanasia, the mice were anaesthetized, and blood was quickly collected from the orbital vessels following the removal of their eyeballs. After that, all mice were euthanized by cervical dislocation. Subsequently, an incision was made from the abdomen of the mice for dissection. In the acute toxicity and in vivo cell migration assays, the heart, liver, spleen, lungs, kidneys, uterus and bilateral ovaries of mice were harvested.

For the immunogenicity study, tail tip blood was collected from each mouse at each time point. Briefly, the mouse tail tip was quickly cut 1–2 mm with sterile surgical scissors, and blood was collected with capillaries.

### Blood test

After incubation for 30 min, the blood samples were further centrifuged to obtain serum. The immune factors and sex hormones were detected using ELISA kits (ab100697, ab282874, SEKM-0034, KGE014, CSB-E06871m) according to the manufacturer’s protocol. For routine blood tests, 20 μL of tail tip blood was collected and assessed within 2 h using a veterinary fully automatic blood cell analyser (BC-5000Vet, Mindray).

### H&E staining and follicle counting

Tissues were fixed with 4% formaldehyde for 24 h, embedded in paraffin and then serially sectioned (5 μm thick) using a microtome. The sections were stained with haematoxylin and eosin to evaluate the intraovarian cellular morphology. Follicle counting was performed manually with an automatic digital slide scanner (Pannoramic MIDI), and only follicles containing the nucleus of an oocyte were counted to avoid any duplicate counting. Follicular stages were classified as follows: primordial follicles surrounded by a single layer of flattened squamous antral granulosa cells; primary follicles surrounded by a single layer of cuboidal granulosa cells; secondary follicles with two or more layers of cuboidal granulosa cells and no visible lumen; and antral follicles with a sinusoidal space with follicular fluid.

### Immunohistochemistry

Briefly, ovarian tissue sections (5 μm thick) were incubated with 5% BSA for 2 h and then incubated with primary antibodies against Ki67 (12202t, CST, 1:600), Cd31 (GB12063, Servicebio, 1:400), Vegf (GB15165, Servicebio, 1:400), Map4k1 (YT2225, Immunoway, 1:300), Cd247 (YT0747, Immunoway, 1:300) or Zap70 (YT4931, Immunoway, 1:300) overnight at 4 °C. After washing, the sections were incubated with the corresponding secondary antibodies (1:200) and horseradish peroxidase. Sections were then washed and stained with 3,3′-diaminobenzidine and counterstained with haematoxylin. Images were obtained with an automatic digital slide scanner (Pannoramic MIDI), and the mean grey values were measured using ImageJ software.

### MSC tracking

For in vivo migration assays of MSCs, MSCs were first fluorescently labelled using a PKH67 Green Fluorescent Cell Linker Mini Kit (MINI67-1KT, Sigma), and after verification by fluorescence microscopy, MSC transplantations were made into mouse ovaries. The mice were later sacrificed on Day 1 and Day 7, and the heart, liver, spleen, lung, kidney, uterus and bilateral ovaries were collected. The tissues were fixed with 4% (w/v) paraformaldehyde, embedded in OCT, stored at − 80 °C, and sectioned after complete freezing. During staining, sections were first washed 3 times with 1 × PBS for 5 min each and then incubated with DAPI staining solution (G1012, Servicebio) for 10 min. Next, the sectioned tissues were washed with 1 × PBS and mounted using antifade mounting medium (G1401, Servicebio). The fluorescence signal was observed under a fluorescence confocal microscope.

### RNA sequencing

Total RNA was extracted from the ovaries of the old-saline-W1, old-saline-W3, old-UC-MSC-W1, old-UC-MSC-W3, old-AD-MSC-W1 and old-AD-MSC-W3 groups. Subsequently, the NEBNext Ultra RNA Library Prep Kit for Illumina was used to construct the libraries for sequencing. The NEBNext Poly(A) mRNA Magnetic Isolation Module kit was used to enrich the poly(A) tailed mRNA molecules from 1 μg of total RNA. The mRNA was fragmented into pieces with an average size of ~ 200 base pairs. First-strand cDNA was synthesized from the mRNA fragments using reverse transcriptase and random hexamer primers, and then, second-strand cDNA was synthesized using DNA polymerase I and RNase H. The ends of the cDNA fragments were repaired, including the addition of a single “A” base, followed by adapter ligation. The products were purified and enriched by PCR to amplify the library DNA. The final libraries were quantified using a KAPA Library Quantification kit (KAPA Biosystems, South Africa) and an Agilent 2100 Bioanalyzer. After RT‒qPCR validation, the libraries were subjected to paired-end sequencing with paired-end 150-base pair reading length on an Illumina NovaSeq sequencer.

### RNA sequencing analysis

The mouse genome version GRCm39 was used as a reference. The sequencing quality was evaluated with FastQC (v0.11.5), and the low-quality data were filtered using NGSQC (v2.3.3). The clean reads were then aligned to the reference genome using HISAT2 (v2.1.0) with default parameters. The processed reads from each sample were aligned using HISAT2 against the reference genome. The gene expression analyses were performed with StringTie (v1.3.3b). DESeq (v1.28.0) was used to analyse the differentially expressed genes (DEGs) between samples. Thousands of independent statistical hypothesis tests were separately conducted on DEGs. A *p* value was then obtained and was corrected by the FDR method. The parameters for classifying significantly DEGs were a fold change of ≥ 2 (|log2FC|≥ 1) in transcript abundance and a *p* value ≤ 0.05.

### Statistical analysis

All statistical analyses were performed using IBM SPSS Statistics 25. Quantitative variables with chi-squared variance were expressed as mean ± SD, and means were compared using Student's t test between two groups or ANOVA followed by Bonferroni post hoc analysis test for comparisons among multiple groups. For data without a Gaussian distribution, the Mann‒Whitney test was used to assess the differences in means. Qualitative variables were compared using the chi-square test. *p value < 0.05 was considered to indicate statistical significance.

## Results

### Establishment of a natural ageing mouse model

**Fig. 2:**
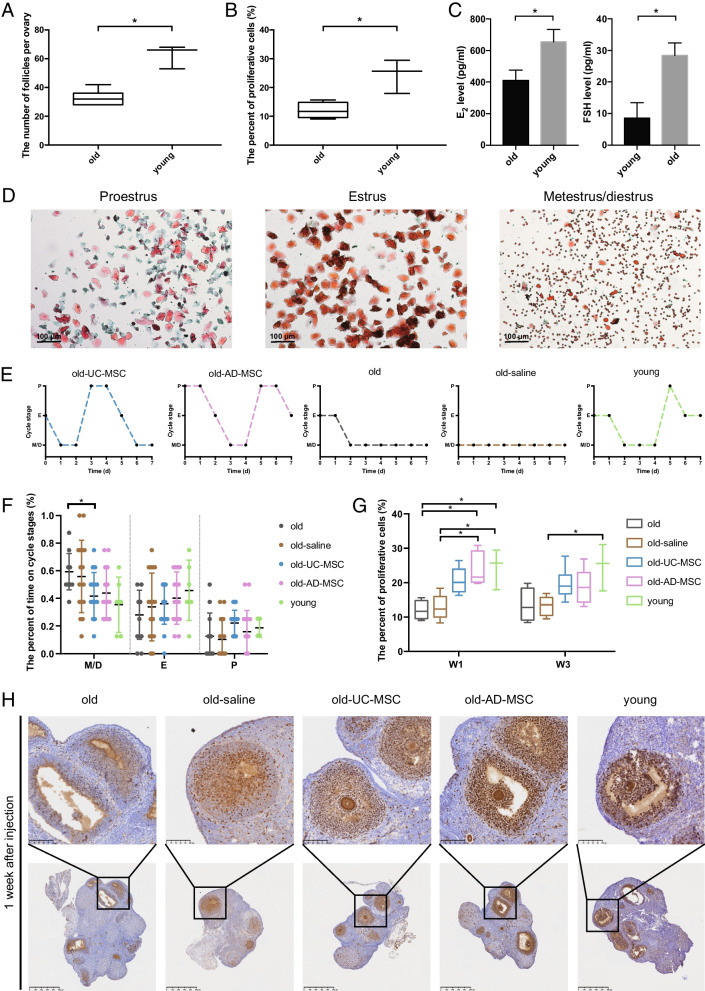
Establishment of a natural ageing mouse model and evaluation of the therapeutic effects of MSCs on ovarian function.** A** The total number of follicles in the ovaries of the old and young groups. **B** The proportion of proliferating cells in the ovaries of the old and young groups. **C** Serum levels of oestradiol (E_2_) and follicle-stimulating hormone (FSH) in the old and young groups. **D** Representative images of vaginal smear staining. **E** Representative line charts of oestrous cycles in the old UC-MSC, old AD-MSC, old, old-saline and young groups. **F** The percent of time spent in the proestrus, estrus and metestrus/diestrus phases among the five groups. **G** The percent of proliferative cells based on Ki67 immunohistochemical staining of ovarian sections over 1 and 3 weeks after MSC transplantation. **H** Representative pathological images of Ki67 immunohistochemical staining of ovarian sections in each group over 1 week after transplantation. The error bars indicate SD. *p < 0.05

C57 mice aged 10–12 months were used as natural ageing model mice (old group, n = 12), and C57 mice aged 4–5 months were used as controls (young group, n = 6). Compared with that of the young group, the reproductive function of the mice in the old group was significantly reduced, as evidenced by the following: (i) the number of follicles was significantly reduced (62.3 follicles per ovary vs. 32.7 follicles per ovary, *p* < 0.05, Fig. 2A), as shown and quantified by H&E staining of the ovaries; (ii) the proportion of proliferating cells in the ovaries was significantly reduced, as shown by immunohistochemistry (from 24.4 to 12.0%, Fig. 2B); and (iii) the levels of oestradiol (E_2_) and follicle-stimulating hormone (FSH) in serum were measured by ELISAs, which showed a significant decrease in E_2_ (from 655.0 pg/ml to 410.3 pg/ml, *p* < 0.05) and elevation in FSH (from 8.6 pg/ml to 28.4 pg/ml, *p* < 0.05) secretion (Fig. 2C).

### Therapeutic effects of AD-MSCs and UC-MSCs

We further included the old-saline group (n = 17), old-AD-MSC group (n = 18) and old-UC-MSC group (n = 18) to observe the beneficial effects of orthotopic MSC transplantation on naturally aged mice and to compare the therapeutic effects of MSCs from different sources. After injection of 10 µl of MSC concentrate containing 3.5*10^5^ MSCs or an equal volume of saline into each ovary, the mice were monitored for 8 consecutive days of the daily oestrous cycle. The mice from all groups were sacrificed over 1 and 3 weeks after transplantation, and their bilateral ovaries were removed for ovarian weight determination, H&E staining and immunohistochemical staining.

(1) Post-transplant oestrous cycle changes

The results revealed that UC-MSC ovarian transplantation improved the oestrous cycle and significantly shortened the metestrus/diestrus phase compared with those of the old group (from 59.4 to 41.7%, *p* < 0.05, Fig. 2D–F). AD-MSC ovarian transplantation also improved the oestrous cycle by decreasing the duration of the metestrus/diestrus phase (from 59.4 to 43.8%) and enhancing the duration of the estrus phase (from 28.1 to 40.3%), although the differences were not statistically significant.

(2) Post-transplant granulosa cell proliferation

The results of Ki67 immunohistochemical staining of ovarian sections suggested that over 1 week after transplantation, the proportion of proliferating cells was significantly increased in the old-AD-MSC group compared with the old and old-saline groups (23.8% vs. 12.0% and 12.9%, *p* < 0.05, Fig. 2G, H, Additional file 1: Figure S2), and the proportion of proliferating cells in the ovaries of the mice in the old-UC-MSC group was slightly increased (20.6%), but there was no significant difference compared with the old control group.

(3) Post-transplant ovarian weight changes

**Fig. 3:**
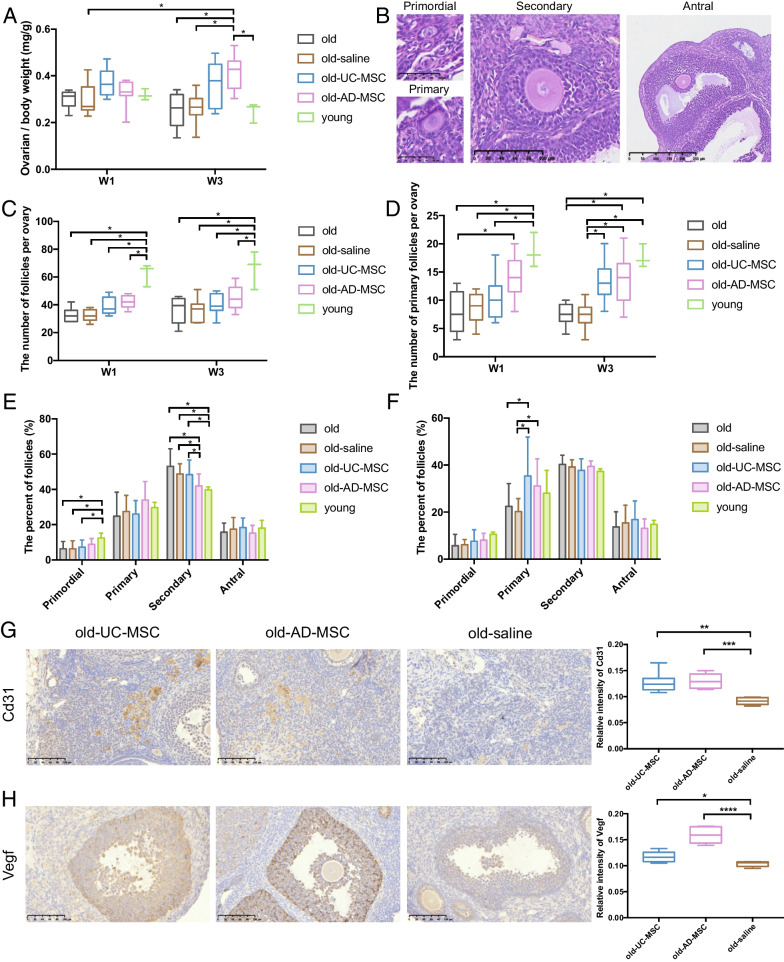
Therapeutic effects of MSCs on follicles and ovarian blood vessels. **A** Bar graph of the ovarian weight of mice in each group, showing a significant increase in ovarian weight over 3 weeks after AD-MSC transplantation. **B** Representative pathological images of primordial, primary, secondary and antral follicles. **C** The total number of follicles in each group indicated that the total follicle number did not increase significantly after MSC transplantation. **D** Number of primary follicles in the ovaries of mice showing a significant increase after UC-MSC and AD-MSC transplantation. **E** Proportion of follicles in each group 1 week after transplantation. **F** Proportion of follicles in each group 3 weeks after transplantation. **G** Representative histochemical staining images of Cd31 in each group and the corresponding bar graph in mouse ovaries over 3 weeks after transplantation. **H** Representative histochemical staining images of Vegf in each group and the corresponding bar graph in mouse ovaries over 3 weeks after transplantation. The error bars indicate SD. *p < 0.05; **p < 0.01; ***p < 0.001; ****p < 0.0001

Over 3 weeks after AD-MSC transplantation, the ovarian weight (normalized by total body weight) of mice increased significantly (0.42 vs. 0.27, *p* < 0.05, Fig. 3A), and the ovarian weight of the mice in the UC-MSC group increased slightly (0.37), but there was no significant difference compared with that of the control group.

(4) Post-transplant follicle count

The results of H&E staining of ovarian sections showed that the total number of follicles in the mouse ovaries did not increase significantly after MSC ovarian transplantation (Fig. 3B, C, Additional file 1: Figure S3). The follicles at each stage were observed and counted, and the results showed that the number of primary follicles increased significantly after UC-MSC and AD-MSC transplantation (Fig. 3D). Over 1 week and 3 weeks after transplantation, the number of primary follicles was significantly increased in the old-AD-MSC group compared with the old group (from 7.8 to 14.1 on W1, from 7.5 to 13.7 on W3, *p* < 0.05), and over 3 weeks after transplantation, the number of primary follicles was significantly increased from 7.3 to 13.4 in the old-UC-MSC group compared with the old-saline group (*p* < 0.05). Subsequent calculation of the proportion of follicles at each stage showed a slight increase in the proportion of primary follicles in the old-AD-MSC group over 1 week after transplantation (Fig. 3E), and over 3 weeks after transplantation, the proportion of primary follicles in mouse ovaries was significantly increased in both the old-UC-MSC (35.4% vs. 22.4% and 20.2%, *p* < 0.05) and old-AD-MSC (31.1% vs. 20.2%, *p* < 0.05) groups (Fig. 3F).

(5) Post-transplant angiogenesis

Histochemical staining of Cd31 and Vegf in the mouse ovaries of both the old-AD-MSC and old-UC-MSC groups indicated a significant increase in angiogenesis in the ovaries of mice 3 weeks after MSC transplantation (*p* < 0.05, Fig. 3G, H). Among them, the increased levels of Cd31 (*p* < 0.001) and Vegf (*p* < 0.0001) in the ovaries of the old-AD-MSC group were significantly greater than those of the old-UC-MSC group.

### Safety of AD-MSC and UC-MSC transplantation

To further evaluate the clinical utility of UC-MSCs and AD-MSCs, we conducted safety assessments on UC-MSCs and AD-MSCs, including tumorigenicity tests, acute toxicity tests, immunogenicity studies and biodistribution studies (tracking MSC migration in vivo).

(1) Tumorigenicity test

**Fig. 4:**
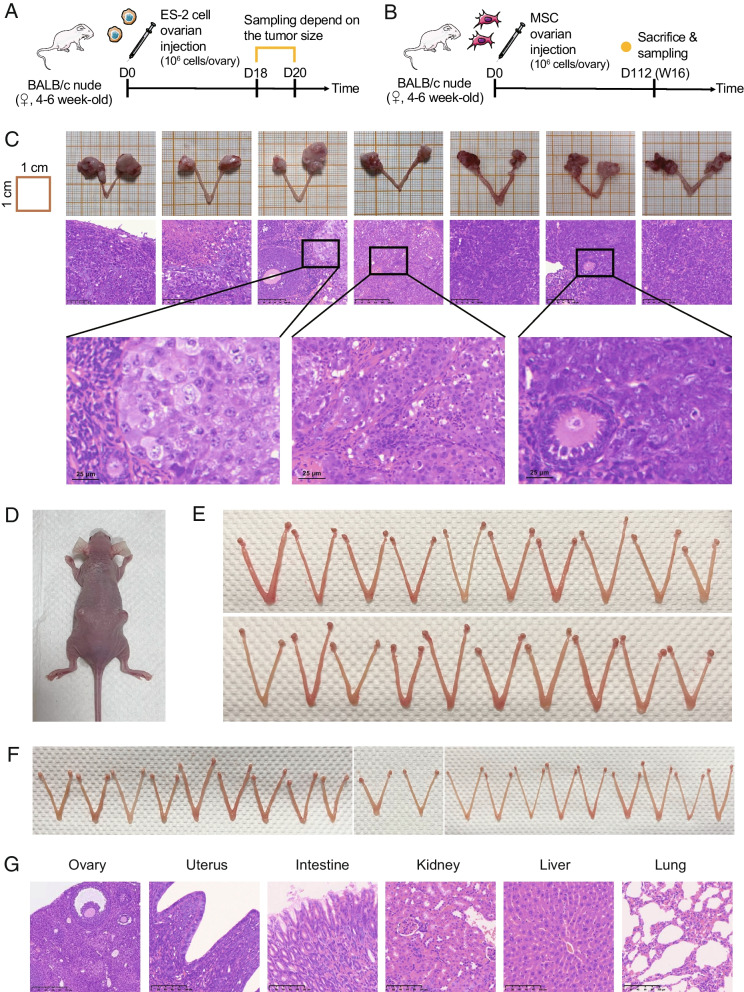
Tumorigenicity test of orthotopic transplantation of MSCs.** A** Flowchart overview of the establishment of an orthotopic xenograft tumour model in mice. **B** Flowchart overview of the evaluation of the tumorigenic potential of MSC transplantation. **C** Photographs and pathological section images of xenograft tumours established in situ. **D** Representative image of mice in the ES-2 injection group. **E** Photographs of ovaries in the AD-MSC injection group. **F** Photographs of ovaries in the UC-MSC injection group. **G** Representative pathological images of ovaries and other organs in the MSC transplantation group

As shown in Fig. 4A, B, we conducted a comparative experiment using BALB/c nude mice aged 4–6 weeks, which were divided into three groups: a positive control group (injected with ES-2 cells, n = 8), an AD-MSC group (injected with AD-MSCs at passage 10, n = 20), and a UC-MSC group (injected with UC-MSCs at passage 10, n = 20). The positive control group was sacrificed on Days 18–20 after transplantation based on tumour size, while the MSC groups were sacrificed after 16 weeks. Tumorigenicity was evaluated using two methods: tumour nodules at the injection site and H&E staining of pathological sections after sampling.

The results showed that all samples from the 8 mice in the positive control group became tumorigenic after bilateral ovarian transplantation of ES-2 cells (tumorigenic rate: 100%, Fig. 4C, D), of which one died on Day 16 after transplantation (postmortem diagnosis showed that it was caused by malignant ascites). No tumorigenesis was observed either in the AD-MSC group or in the UC-MSC group after 16 weeks (tumorigenic rate: 0%, Fig. 4E, F). H&E staining of ovary sections confirmed the above results (Additional file 1: Figure S4). Additionally, H&E staining of mouse organs (intestine, uterus, spleen, kidney, liver, and lung) showed that mice in both the AD-MSC and UC-MSC groups were healthy with no tumorigenesis (Fig. 4G). During the follow-up process, no obvious abnormalities in the body weight, appearance, behaviour, or health status of mice were found in the AD-MSC group or the UC-MSC group.

In summary, the tumorigenicity was 100% (8/8) when ovarian cancer cells were injected into the ovaries of BALB/c nude mice, confirming that BALB/c nude mice are highly susceptible to ovarian cancer cells. When MSCs were injected into the ovaries of BALB/c nude mice, tumorigenicity was 0%. Therefore, AD-MSCs and UC-MSCs were not tumorigenic when orthotopically transplanted into the ovaries of BALB/c nude mice (*p* < 0.0001).

(2) Acute toxicity test

For the acute toxicity test, 4-week-old C57BL/6 mice were used and divided into a normal control group (n = 6) and a UC-MSC transplantation group (n = 12). The mice were weighed on the day after the initiation of the experiment, sequentially on Day 7 and Day 14, and each organ sampling was completed on Day 14. Whether the mice possessed acute toxic reactions to UC-MSC transplantation in vivo was determined by observing general indices (appearance, behaviour, mental status, etc.), mortality, and weight changes and anatomical and pathological examination of each organ during the experimental period. The results indicated that both groups of mice survived and were in good mental condition, with no significant abnormalities observed in their appearance or behaviour. In terms of weight changes, the mice in both groups showed a tendency to increase in weight, and the mean weight fluctuated from 16.9–18.7 g in the control group to 16.3–18.2 g in the treatment group, with no significant difference between the groups (*p* > 0.05, Additional file 1: Figure S5A). Histological anatomy (volume, colour, texture) and pathological findings of each organ did not show significant abnormalities in either group (Additional file 1: Figure S5B).

In summary, the results showed that after the transplantation of UC-MSCs, the mice were in good general condition without any accidental death or significant abnormalities, and there were no significant differences in their body weight growth compared with the control group. Therefore, it can be concluded that there was no significant acute toxic reaction after UC-MSC transplantation into mice. Similarly, the mice in the AD-MSC group were generally in good condition without any accidental deaths, and no significant abnormalities were found in the histological and pathological examinations, which confirmed that AD-MSCs had no acute toxic reactions.

(3) Immunogenicity study

**Fig. 5:**
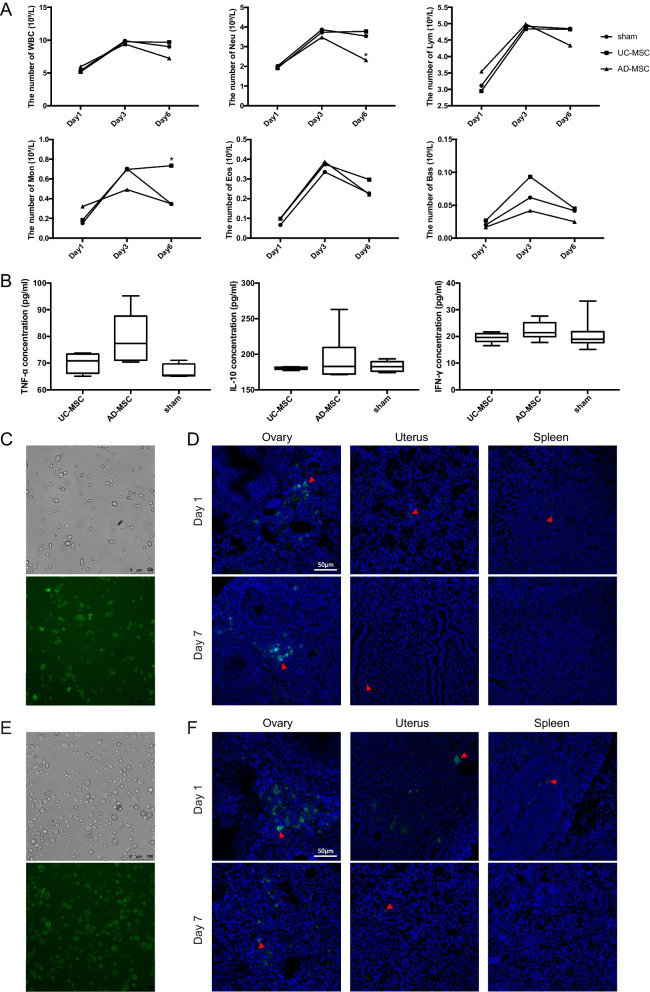
Immunogenicity study and biodistribution study of MSC transplantation.** A** The number of WBC (white blood cells), Neu (neutrophils), Lym (lymphocytes), Mon (monocytes), Eos (eosinophils) and Bas (basophils) in the mouse serum on Day 1, Day 3 and Day 6. **B** The concentration of immune molecules (IL-10, TNF-α, IFN-γ) in the serum of mice on Day 6. **C** Images of UC-MSCs under bright field (top) and green fluorescence (bottom) after PKH67 fluorescent dye labelling. **D** Representative images of ovary, uterus and spleen sections showing UC-MSC distribution on Day 1 and Day 7 after injection. **E** Images of AD-MSCs under bright field (top) and green fluorescence (bottom) after PKH67 fluorescent dye labelling. **F** Representative images of ovary, uterus and spleen sections showing AD-MSC distribution on Day 1 and Day 7 after injection. The error bars indicate SD. *p < 0.05

For the immunogenicity study, 9-month-old C57BL/6 mice were used and divided into three groups: the sham group (injected with PBS, n = 6), AD-MSC transplantation group (injected with AD-MSCs, n = 6) and UC-MSC transplantation group (injected with UC-MSCs, n = 6). Tail tip blood was collected on Days 1, 3 and 6 after injection. Mice were sacrificed on Day 6, and the material was harvested. The immunogenicity of MSCs was determined by measuring the blood routine on Days 1, 3 and 6 and the concentration of immune molecules (IL-10, TNF-α, IFN-γ) in the serum of mice on Day 6. The results showed that there were no differences in the white blood cell (WBC), lymphocyte (Lym), eosinophil (Eos) and basal (basophil) counts among the groups. On Day 6, a difference in the content of monocytes (Mon) appeared between the UC-MSC group and sham group, with the UC-MSC group having higher levels than the sham group (0.35*10^9^/L vs. 0.74*10^9^/L, *p* < 0.05) (Fig. 5A). In terms of immune molecules, there were no significant differences in serum TNF-α, IL-10 and IFN-γ concentrations among the groups (Fig. 5B).

In summary, the data revealed that the number of immune cells did not significantly increase in the majority of mice after MSC transplantation, and there were no significant increases in the expression of immune molecules in mice after MSC transplantation, thus indicating the low immunogenicity of both AD-MSCs and UC-MSCs.

(4) Biodistribution study

To assess MSC migration in mice, we selected 8-week-old C57BL/6 mice, which were orthotopically injected with PKH67 fluorescent dye-labelled UC-MSCs (n = 6) or AD-MSCs (n = 6). Three mice from each group were sacrificed on Day 1, and the remaining three were sacrificed on Day 7 post-transplantation. Tissue sections were prepared from each organ to observe the distribution of MSCs in mice after transplantation. Our results showed that on Day 1, PKH67-labelled MSCs were mainly detected in the ovaries, with a small amount found in the uterus and spleen (Fig. 5C–F). On Day 7, MSCs were still mainly concentrated in the ovaries, with a small amount found in the uterus but no signal detected in the spleen. PKH67-labelled MSCs were not detected in any other organs (brain, intestine, heart, kidney, liver, lung, stomach) on either Day 1 or Day 7 (Fig. 5D, F, Additional file 1: Figure S6).

Overall, the results of the biodistribution study revealed that fluorescence was predominantly enriched in the ovaries, with lower levels observed in the uterus and spleen, indicating a low degree of nondeterministic distribution of MSCs. Taken together, the results suggest that both AD-MSCs and UC-MSCs possess high safety profiles when transplanted into mouse ovaries.

### RNA sequencing analysis of MSC transplantation

**Fig. 6:**
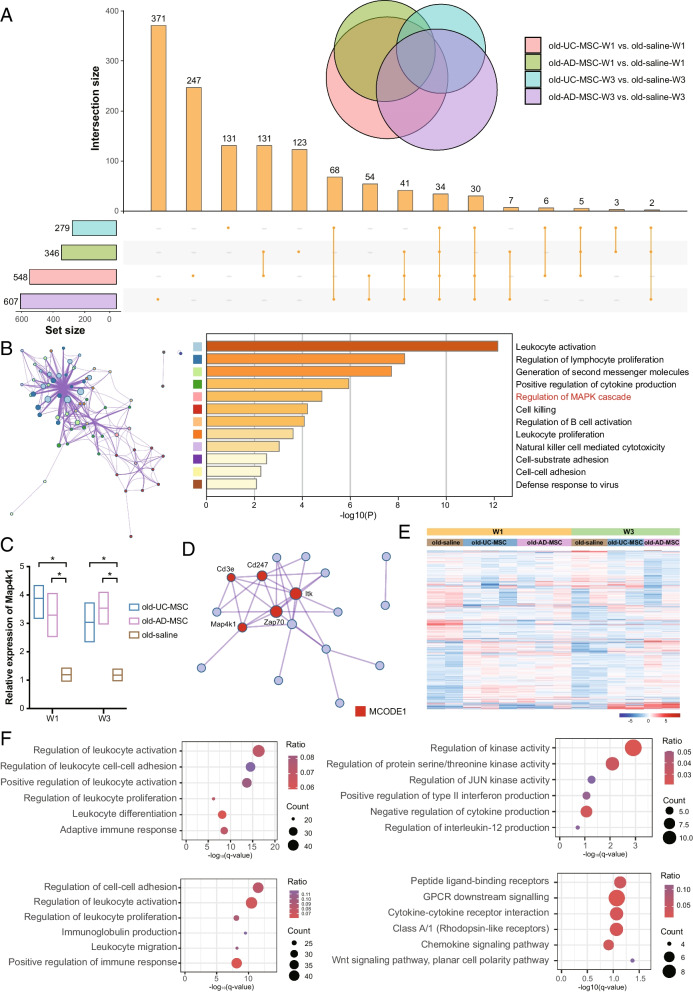
Transcriptome changes in mouse ovaries after MSC transplantation. **A** The number of upregulated genes in each group after MSC transplantation. Among them, 34 genes were shared among the groups. **B** Bar graph of the top 12 enriched terms across the 34 shared genes, coloured by *p* values. Network of enriched terms (left) coloured by cluster ID, where nodes that share the same cluster ID are typically close to each other. **C** Relative expression levels of Map4k1, a key gene in the MAPK pathway, in each group. The error bars indicate SD. *p < 0.05. **D** Protein‒protein interaction network and MCODE components identified through analysis of the 34 shared genes. **E** Heatmap of DEGs in each group. **F **Bubble graphs of enriched terms across unique up-regulated genes in each group. Old-UC-MSC-W1 versus old-saline-W1 (top left, 337 genes), old-AD-MSC-W3 versus old-saline-W3 (bottom left, 473 genes), old-UC-MSC-W3 versus old-saline-W3 (top right, 135 genes), old-AD-MSC-W1 versus old-saline-W1 (bottom right, 145 genes)

For each sample, we sequenced an average of 44.5 million raw reads (ranging from 40.7 to 49.4 million reads), with mapping rates of approximately 95.2% (ranging from 94.9 to 95.5%, sequencing quality statistics in Additional file 2: Table 1). An average of 34,921 genes were detected. We compared the upregulated genes in each group, and 34 shared genes were discovered (Fig. 6A, Additional file 2: Table 2). Enrichment analysis of these genes revealed that the regulation of the MAPK cascade was enhanced after UC-MSC and AD-MSC transplantation, whether for a short-term (1 week) or long-term (3 weeks) period (Fig. 6B), with a significant increase in Map4k1 expression (Fig. 6C). Protein‒protein interaction network and MCODE component analyses were further performed, and a network mainly composed of Cd3e, Cd247, Map4k1, Zap70 and Itk was identified (Fig. 6D) and supported by immunohistochemical experiments (Additional file 1: Figure S7). These genes might play an indelible role in improving ovarian function after MSC transplantation. Next, unique upregulated genes in each group were analysed to identify different mechanisms of UC-MSC and AD-MSC treatment on ovarian ageing in mice (Fig. 6E, F). Over 3 weeks after AD-MSC transplantation, 473 unique upregulated genes were discovered, while only 135 unique upregulated genes were discovered for the UC-MSC transplantation group after 3 weeks. This finding suggested that after AD-MSC transplantation, changes in ovarian function were more intense, with enrichment in cell‒cell adhesion and positive regulation of the immune response. Moreover, short-term effects were mainly concentrated in kinase activity, GPCR signalling, chemokine signalling and the Wnt signalling pathway, while long-term effects were enriched in the activation of immune function. This result suggested differences in the mechanism of short-term and long-term effects.

## Discussion

Incremental studies show that MSCs possess excellent therapeutic potential for a variety of clinical diseases that have proven refractory to traditional treatment modalities. Ovarian ageing is one of the major concerns impacting female fertility, and MSC-based therapy has made remarkable progress in this area. Previous studies examining the therapeutic use of stem cells in the ovary have primarily employed tail vein injection as the route of administration, with most of these studies utilizing premature ovarian failure models as the study subjects [19, 20]. In contrast, the orthotopic transplantation approach adopted in this study delivered MSCs more directly to the site of action, thereby circumventing the need for MSC migration along the way, and was expected to produce more efficacious therapeutic effects on the ovary. In this study, we utilized two types of tissue-derived MSCs, both of which were administered via orthotopic transplantation, and their efficacy and safety were validated.

Ding et al. reported a significant efficacy at 4 weeks after orthotopic transplantation of mice using amniotic-origin MSCs [21]. This finding was evidenced by an increase in the number of follicles at all stages, elevated levels of E_2_, FSH and anti-Mullerian hormone, and an increase in granulosa cell proliferation. The results of our study show an earlier onset of action of MSCs compared to the results of this study and further demonstrate the difference in efficacy of MSCs from different sources. Both AD-MSC and UC-MSC transplantation improved ovarian function in ageing mice, with AD-MSCs being more effective than UC-MSCs. However, previous studies in rats treated with tail vein infusion of MSCs for natural ovarian ageing appeared to show an earlier improvement (over 2 weeks after transplantation) [22]. This difference may be related to the amount of MSCs used, as they injected 1*10^7^ UC-MSCs into each rat. Although two other tail vein dosing experiments using 5*10^5^ cells per injection showed that multiple doses of MSC injection led to earlier ovarian improvement compared to ovariectomized rats [23, 24], we suggest that a single dose is still the better choice for clinical practice due to patient compliance and the benefit/risk ratio. In addition, the improvement effects on hormone levels after transplantation were not significant in this study, which may indicate the insensitivity of hormone levels to MSCs and was consistent with previous research findings [25, 26].

Based on the validation of the efficacy of MSC therapy, we experimentally validated the safety of orthotopic transplantation of MSCs. Since orthotopic transplantation into the ovary is a relatively new transplantation modality, previous studies have not extensively investigated this area. In this study, experiments were conducted to assess tumorigenicity, acute toxicity, immunogenicity and migration in vivo. The tumorigenicity test is an important component of the safety testing of cell therapy methods, which is commonly needed in the guidance on drug filing, and the results of this study suggest that MSCs are not tumorigenic. Moreover, no significant acute toxic reactions were observed in mice after MSC transplantation. The above results are consistent with the results of two trials that used MSCs for the treatment of human ovarian diseases [14, 15]. Moreover, the study by Wang et al. conducted long-term observation after MSC transplantation for tumorigenicity assessment. Although no positive control group was established, their results also indicated the safety of orthotopic transplantation of MSCs into the ovary [27].

This study demonstrates the low immunogenicity of MSCs after transplantation into mice. Zhu et al. also raised concerns about immunogenicity after MSC transplantation in an experiment using cyclophosphamide to induce ovarian damage in rats [28]. Observations after sectioning of each tissue showed no significant difference compared to those of the PBS-injected control group, but one rat in the MSC tail vein injection group developed a brain infarct. Although the article did not provide a mechanistic discussion of this phenomenon, it may suggest a safety issue with direct blood injection of MSCs.

After orthotopic transplantation of MSCs, a small amount of nondeterministic distribution of MSCs was indicated for both short-term (Day 1) and long-term (Day 7) observation. The results from Yoon et al. also suggested that human MSCs were mainly present in the ovary, uterus and spleen one day after transplantation into mice, while their presence could only be detected in the spleen after 7 days [19]. In contrast, studies using the same MSC-labelled tracer method showed that MSCs were mainly localized in the perivascular area, in some cases close to primary follicles, and even in contact with granulosa cells and that the number of cells tended to increase after 7 and 14 days of injection [29]. This discrepancy may be caused by the different sources of MSCs used. The results of Su et al. showed that after the use of soluble collagen scaffolds, MSCs can still be detected more strongly within 2 weeks [20]. This finding suggests that the duration of MSC action in vivo can be increased by means of composite materials. However, the results of previous studies suggested that fluorescently labelled MSCs from in vitro sources did not survive for more than one month in vivo. In addition, although some studies have shown that the majority of MSCs injected into the tail vein were distributed in the ovary and uterus, it was found that orthotopically injected UC-MSCs were detected only in the ovary and uterus, while UC-MSCs injected into the tail vein were also found in the kidney, liver, and lung in a comparative study of orthotopic injection versus tail vein injection of MSCs [28].

## Conclusions

This study successfully established a mouse model of natural ovarian ageing and demonstrated that orthotopic transplantation of MSCs from different sources into the ovary improved the ovarian function of ageing mice. Additionally, the effect of AD-MSCs was superior to that of UC-MSCs. Safety validation experiments confirmed that both AD-MSCs and UC-MSCs were not tumorigenic, with no acute toxic reactions, low immunogenicity, and a small amount of nondeterministic distribution. Furthermore, the mechanisms underlying the long-term and short-term effects after MSC transplantation differed, yet both led to an enhancement of the MAPK cascade. Collectively, orthotopic transplantation of MSCs has significant efficacy and high safety in the treatment of ovarian ageing.

### Supplementary Information

**Additional file 1:** Supplementary figures.

**Additional file 2:** Tables for RNA sequencing analysis.

## Data Availability

The raw RNA sequencing data of mouse ovaries are available at the Genome Sequence Archive (CRA012256). Shared URL: https://bigd.big.ac.cn/gsa/browse/CRA012256.

## Abbreviations

MSC: Mesenchymal stem cell
UC-MSC: Umbilical cord-derived mesenchymal stem cell
AD-MSC: Adipose tissue-derived mesenchymal stem cell
GMP: Good manufacturing practice
SPF: Specific pathogen-free
ELISA: Enzyme-linked immunosorbent assay
PBS: Phosphate‑buffered saline
FITC: Fluorescein isothiocyanate
BSA: Bovine serum albumin
OCT: Optimum cutting temperature
DAPI: 4′,6-Diamidino-2phenylindole
RT‒qPCR: Quantitative real-time polymerase chain reaction
DEG: Differentially expressed gene
E_2_: Oestradiol
FSH: Follicle-stimulating hormone
