# Supplementary material for: Efficacy and safety of mesenchymal stem cell therapy for ovarian ageing in a mouse model
Source: Stem Cell Res Ther. 2024 Apr 3;15:96. doi: 10.1186/s13287-024-03698-0 (PMC10988907; doi:10.1186/s13287-024-03698-0)
Supplement: Supplementary file 1 — Additional file 1: Supplementary figures. [file 13287_2024_3698_MOESM1_ESM.docx]

**Supplementary Figures**


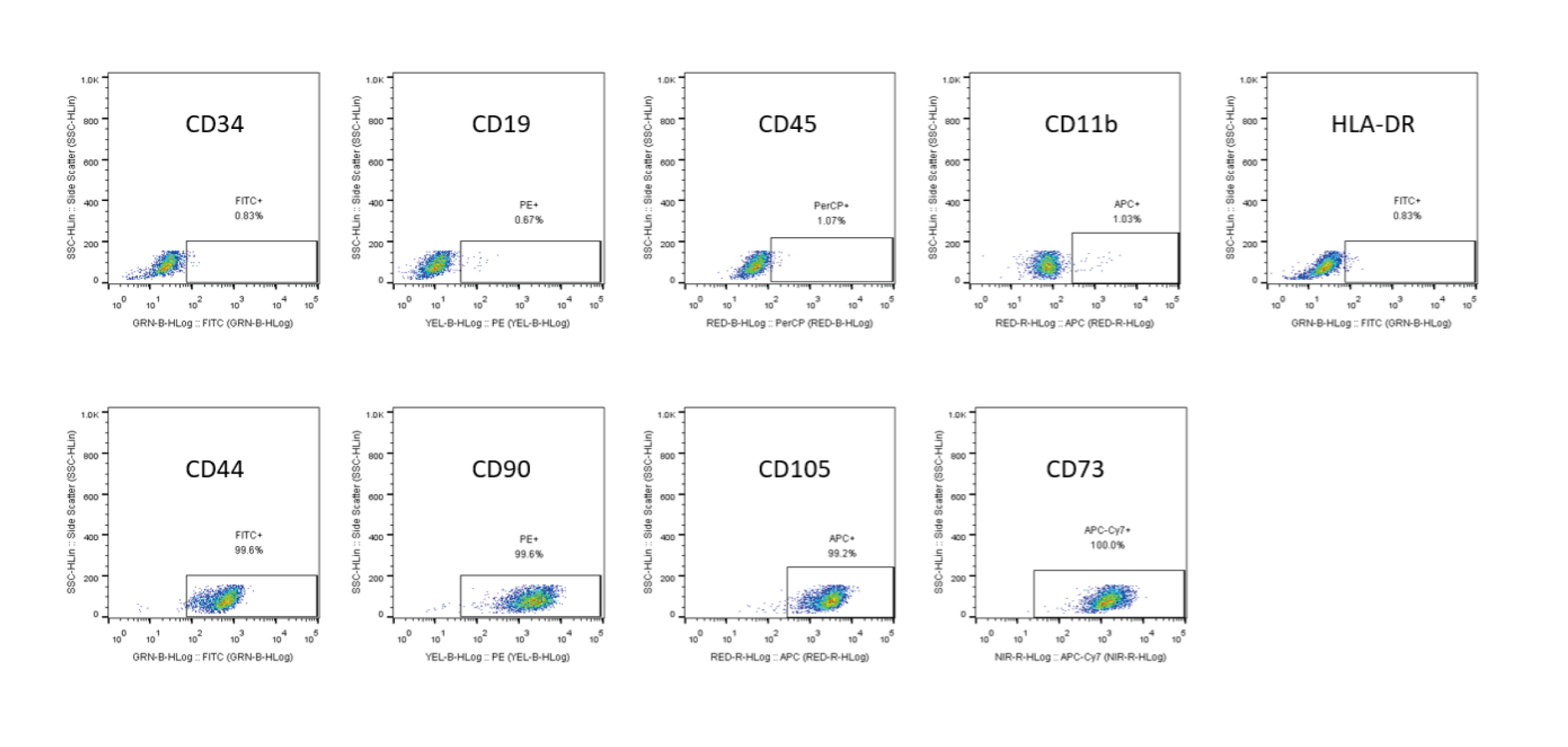


**Figure S1. The results of flow cytometry detection of MSC surface markers.**


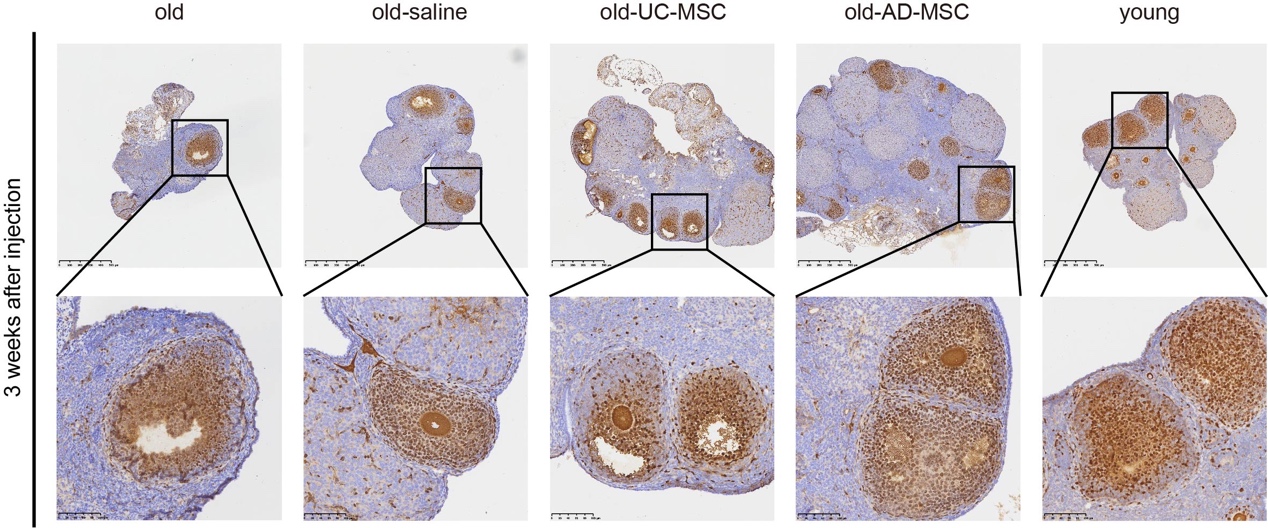


**Figure S2. Representative images of Ki67 immunohistochemical staining in mouse ovaries at 3 weeks after MSC transplantation.**


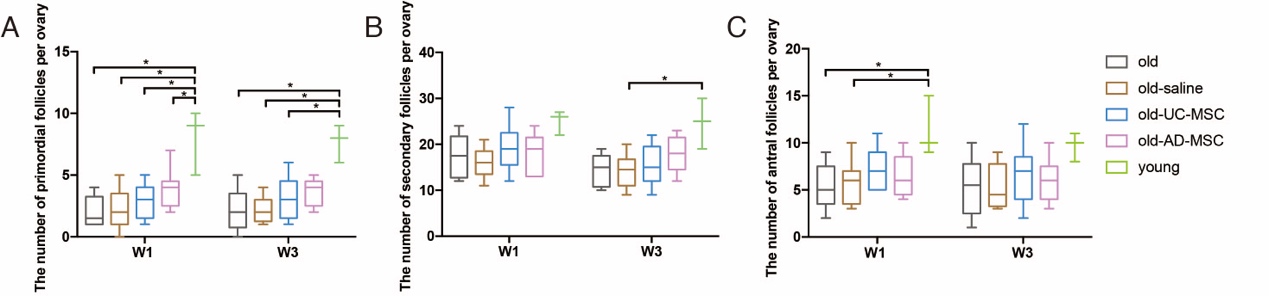


**Figure S3. The number of primordial (A), secondary (B) and antral follicles (C) at 1 week and 3 weeks after MSC transplantation.**

The error bars indicate SD. *p < 0.05.


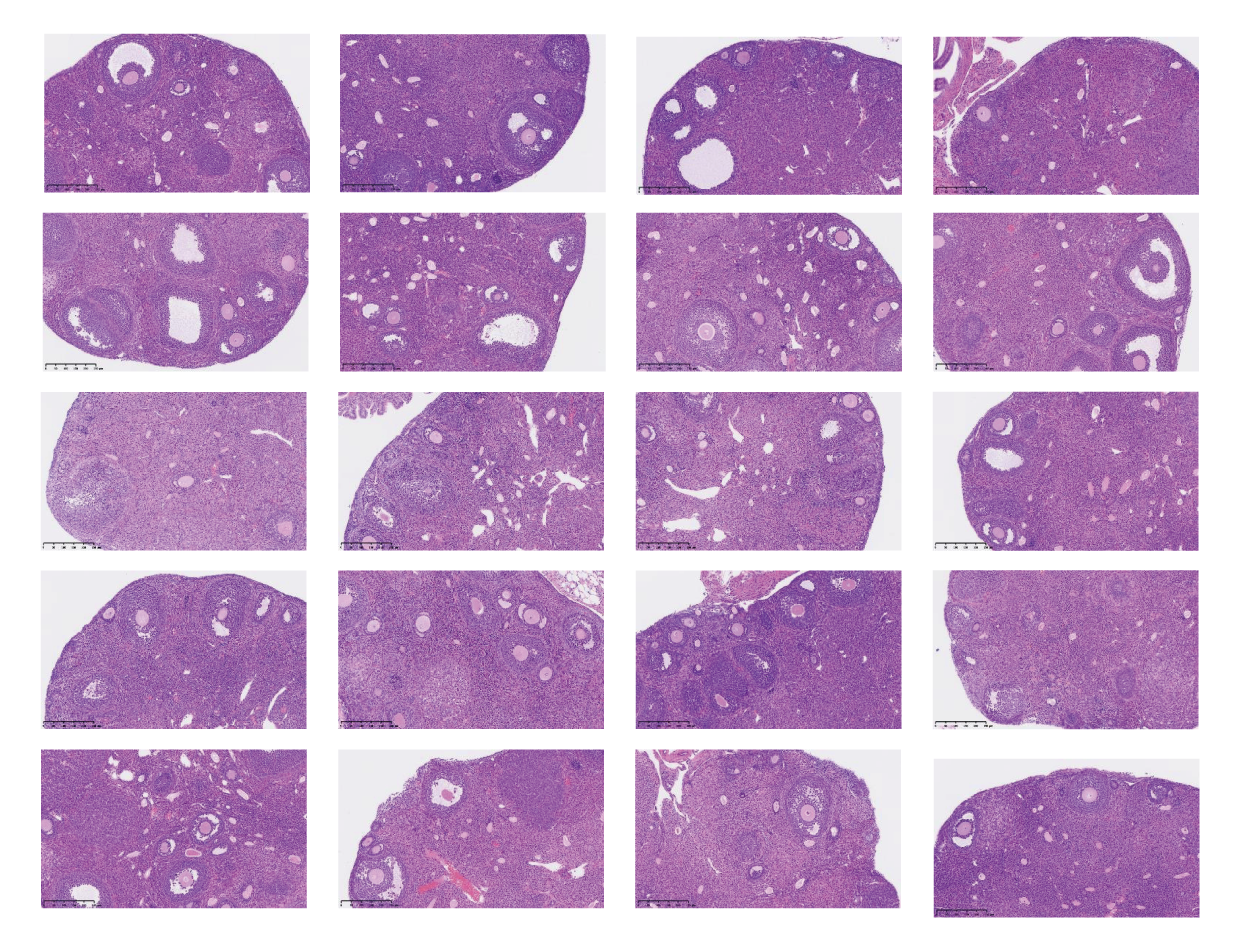


**Figure S4. Pathological section images of mouse ovaries after MSC injection in tumorigenicity test.**


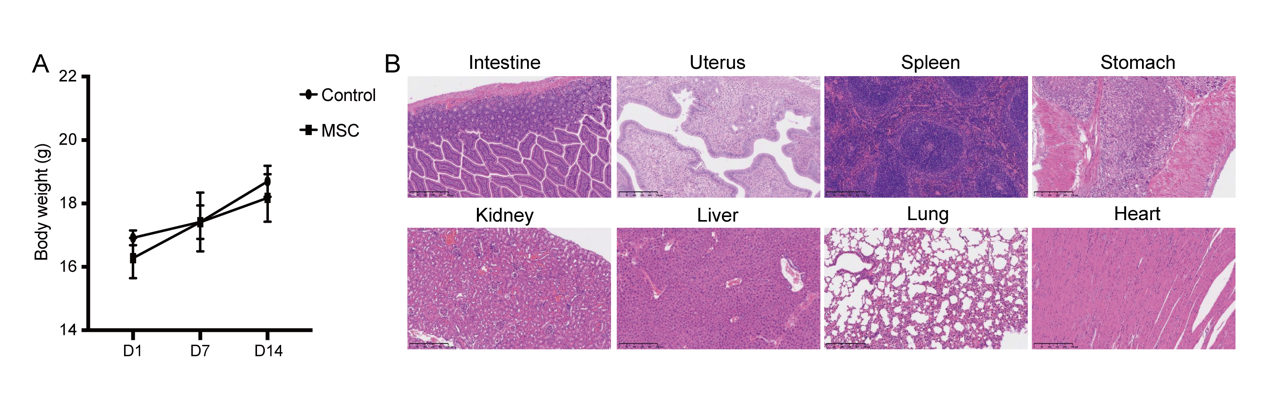


**Figure S5. The results of acute toxicity test.**

(A) The weight changes of mice in both groups trended towards weight gain, with no significant difference between the groups (p > 0.05). (B) Representative images of pathological findings in mouse organs from the MSC group.


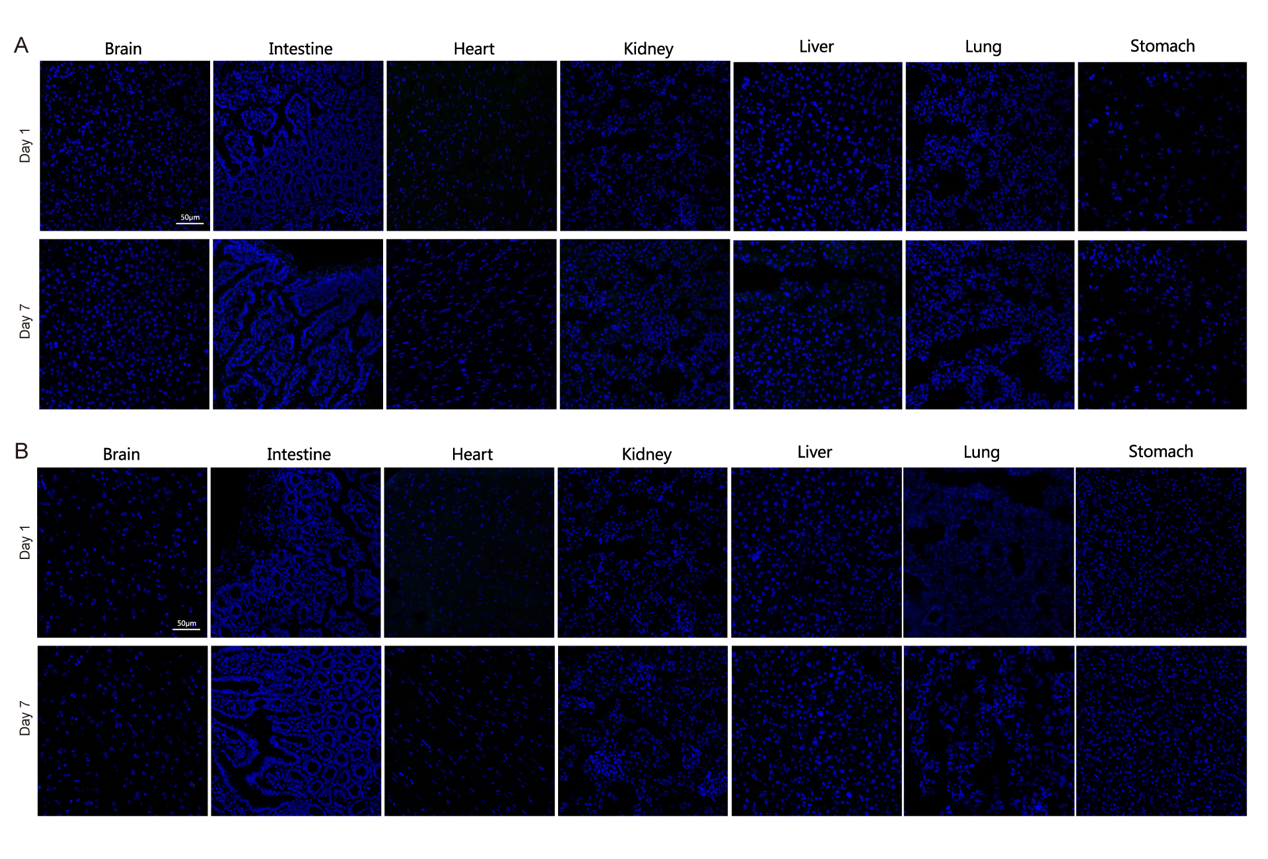


**Figure S6. No distributions of UC-MSCs (A) or AD-MSCs (B) were found in other organs (brain, intestine, heart, kidney, liver, lung, stomach) on Day 1 or Day 7 after injection.**


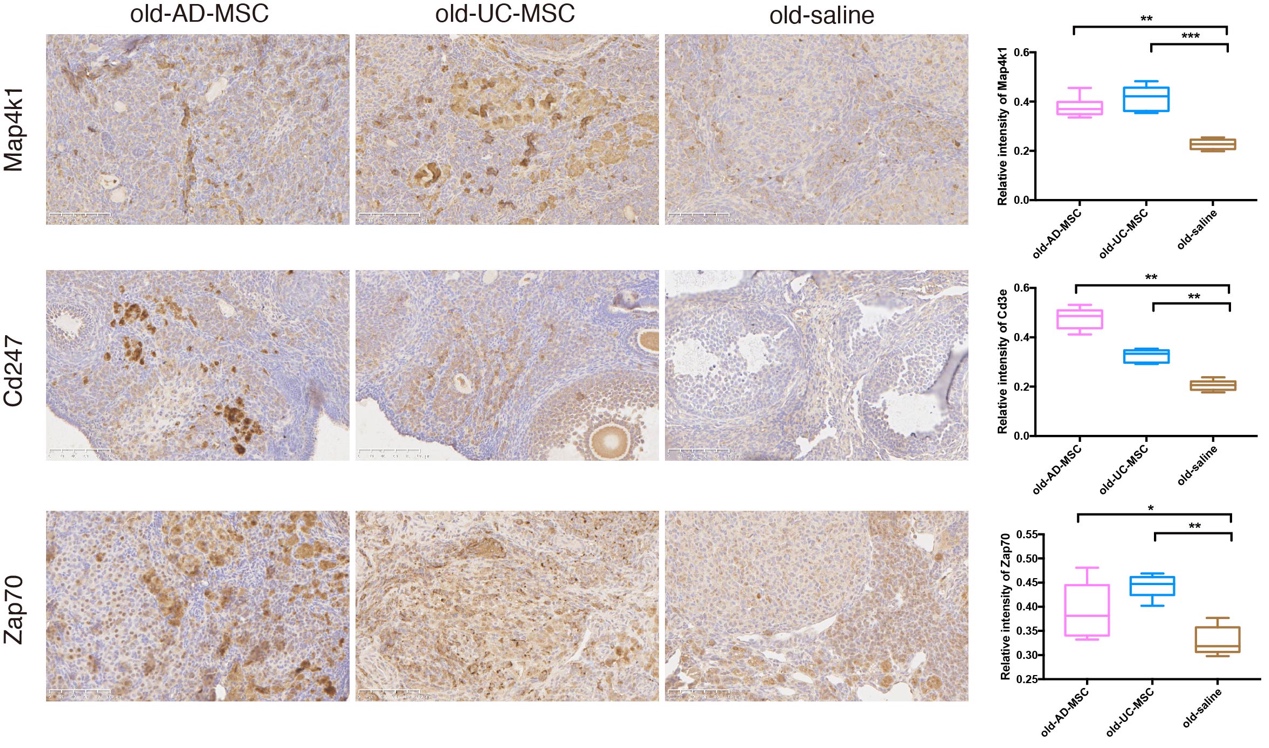


**Figure S7. Immunohistochemical experiments revealed that Map4k1, Cd3e and Zap70 increased after MSC transplantation.**
