# Supplementary material for: Efficacy and safety of mesenchymal stem cell therapy for ovarian ageing in a mouse model
Source: Stem Cell Res Ther. 2024 Apr 3;15:96. doi: 10.1186/s13287-024-03698-0 (PMC10988907; doi:10.1186/s13287-024-03698-0)
Supplement: Supplementary file 2 — Additional file 2: Tables for RNA sequencing analysis. [file 13287_2024_3698_MOESM2_ESM.docx]

**Supplementary Tables**

**Supplementary Table 1. RNA sequencing quality of mouse ovaries.**

| **Sample** | **Raw reads number** | **Clean reads number** | **Q30 (%)** | **Uniquely mapped reads (%)** | **Gene number** | **Group** |
| --- | --- | --- | --- | --- | --- | --- |
| old_w1_1 | 41,482,148 | 40,073,140 | 92.78 | 36,777,844 (95.29%) | 35,276 | old-saline (W1) |
| old_w1_2 | 45,511,210 | 44,255,130 | 92.68 | 40,823,644 (95.11%) | 31,992 |  |
| old_w3_1 | 49,247,544 | 47,533,084 | 92.59 | 43,614,195 (94.97%) | 36,101 | old-saline (W3) |
| old_w3_2 | 49,376,002 | 47,674,866 | 92.9 | 43,764,929 (95.17%) | 33,576 |  |
| usc_w1_1 | 44,032,466 | 42,487,210 | 92.27 | 39,074,247 (95.25%) | 34,679 | old-UC-MSC (W1) |
| usc_w1_2 | 41,934,190 | 40,687,180 | 92.53 | 37,460,079 (95.22%) | 32,268 |  |
| usc_w1_3 | 43,792,320 | 42,329,270 | 92.18 | 38,910,871 (95.44%) | 31,821 |  |
| usc_w3_2 | 43,502,600 | 42,333,984 | 93.18 | 39,014,673 (95.13%) | 35,880 | old-UC-MSC (W3) |
| usc_w3_3 | 40,688,466 | 39,321,818 | 92.55 | 36,347,755 (95.47%) | 31,955 |  |
| asc_w1_1 | 43,411,656 | 41,987,242 | 92.84 | 38,756,074 (95.21%) | 30,755 | old-AD-MSC (W1) |
| asc_w1_2 | 43,259,542 | 41,905,992 | 92.69 | 38,666,815 (95.38%) | 28,912 |  |
| asc_w1_3 | 44,797,546 | 43,156,310 | 92.4 | 39,812,327 (95.43%) | 34,255 |  |
| asc_w3_1 | 49,205,522 | 47,312,774 | 93.02 | 43,316,798 (94.92%) | 33,260 | old-AD-MSC (W3) |
| asc_w3_3 | 43,330,724 | 42,027,666 | 92.98 | 38,654,900 (95.25%) | 36,986 |  |

**Supplementary Table 2. The list of the 34 shared genes.**

| **No.** | **Ensemble ID** | **Gene symbol** |
| --- | --- | --- |
| 1 | ENSMUSG00000022439 | Parvg |
| 2 | ENSMUSG00000040247 | Tbc1d10c |
| 3 | ENSMUSG00000076617 | Ighm |
| 4 | ENSMUSG00000027368 | Dusp2 |
| 5 | ENSMUSG00000039264 | Gimap3 |
| 6 | ENSMUSG00000018654 | Ikzf1 |
| 7 | ENSMUSG00000028071 | Sh2d2a |
| 8 | ENSMUSG00000054342 | Kcnn4 |
| 9 | ENSMUSG00000026832 | Cytip |
| 10 | ENSMUSG00000021298 | Gpr132 |
| 11 | ENSMUSG00000041202 | Pla2g2d |
| 12 | ENSMUSG00000018168 | Ikzf3 |
| 13 | ENSMUSG00000024670 | Cd6 |
| 14 | ENSMUSG00000044309 | Apol7c |
| 15 | ENSMUSG00000029275 | Gfi1 |
| 16 | ENSMUSG00000037337 | Map4k1 |
| 17 | ENSMUSG00000027863 | Cd2 |
| 18 | ENSMUSG00000020395 | Itk |
| 19 | ENSMUSG00000056290 | Ms4a4b |
| 20 | ENSMUSG00000037318 | Traf3ip3 |
| 21 | ENSMUSG00000024164 | C3 |
| 22 | ENSMUSG00000026117 | Zap70 |
| 23 | ENSMUSG00000030789 | Itgax |
| 24 | ENSMUSG00000056498 | Tmem154 |
| 25 | ENSMUSG00000026875 | Traf1 |
| 26 | ENSMUSG00000031779 | Ccl22 |
| 27 | ENSMUSG00000048251 | Bcl11b |
| 28 | ENSMUSG00000032093 | Cd3e |
| 29 | ENSMUSG00000029417 | Cxcl9 |
| 30 | ENSMUSG00000020437 | Myo1g |
| 31 | ENSMUSG00000033220 | Rac2 |
| 32 | ENSMUSG00000005763 | Cd247 |
| 33 | ENSMUSG00000024610 | Cd74 |
| 34 | ENSMUSG00000041642 | Kif21b |
